# Treatment of Wounds

**Published:** 1916-05

**Authors:** 


**Treatment of Wounds.** H. Bourgeois, Prog. Med., Feb. 5, 1916. Otologists have long recognized the obstinacy of the bacillus pyocyaneus and its tendency to produce a perichondritis. It produces a characteristic odor preceding the color by several days. It can be satisfactorily combatted by balsam of Peru, either spread on the surface or introduced into fistulae on bits of gauze. It should be employed in very small quantity and renewed every day or every other day. It is encountered also in war surgery. An extensive wound should be treated, not with the pure balsam but with the following: essence of marjorna 0.25, essence of verbena 0.25,
following: essence of marjoram 0.25, essence of verbena, 0.25,

The bacillus pyocyaneus finds itself quite at its ease (Quaint non-technical expressions like this are common in French medical writings and impress their lesson on the reader much better than the more dignified expressions used by most English and American writers) in wounds treated by hydrogen peroxid but for almost all other wound infections, the latter continues to enjoy a well deserved vogue. While one can wash a wound with hydrogen peroxid it cannot be used for a fixed dressing for the oxygen soon disappears. For this purpose, we have long used perborate of sodium which yields its oxygen gradually in contact with the bodily liquids. This action lasts for 12-18 hours. The action is not antiseptic alone, indeed, the first dressings increase the flow of pus and surgeons are found who refuse to employ it on the ground that it increases the infection. But, at the end of 48 hours, the pus has lost its fetor if it was present at the beginning and the secretion gradually becomes less purulent and changes to one of lymph. Granulations become healthy and are not redundant so that epidermization is not interfered with and it is unnecessary to use silver nitrate. The objections to sodium perborate are that it cannot be sterilized, sometimes produces pain and is sometimes irritating to the skin. The same objections apply to hydrogen peroxid and, indeed, the solutions furnished are sometimes simply solutions of sodium perborate. Sodium perborate should not be used on aseptic wounds or on a fresh wound which can readily be sterilized, nor in the visceral cavities nor joints.

